# Peroxisomal Alanine: Glyoxylate Aminotransferase AGT1 Is Indispensable for Appressorium Function of the Rice Blast Pathogen, *Magnaporthe oryzae*

**DOI:** 10.1371/journal.pone.0036266

**Published:** 2012-04-27

**Authors:** Vijai Bhadauria, Sabine Banniza, Albert Vandenberg, Gopalan Selvaraj, Yangdou Wei

**Affiliations:** 1 Department of Biology, University of Saskatchewan, Saskatoon, Saskatchewan, Canada; 2 Crop Development Centre, University of Saskatchewan, Saskatoon, Saskatchewan, Canada; 3 Plant Biotechnology Institute, National Research Council of Canada, Saskatoon, Saskatchewan, Canada; Seoul National University, Republic of Korea

## Abstract

The role of β-oxidation and the glyoxylate cycle in fungal pathogenesis is well documented. However, an ambiguity still remains over their interaction in peroxisomes to facilitate fungal pathogenicity and virulence. In this report, we characterize a gene encoding an alanine, glyoxylate aminotransferase 1 (AGT1) in *Magnaporthe oryzae*, the causative agent of rice blast disease, and demonstrate that *AGT1* is required for pathogenicity of *M. oryzae*. Targeted deletion of *AGT1* resulted in the failure of penetration via appressoria; therefore, mutants lacking the gene were unable to induce blast symptoms on the hosts rice and barley. This penetration failure may be associated with a disruption in lipid mobilization during conidial germination as turgor generation in the appressorium requires mobilization of lipid reserves from the conidium. Analysis of enhanced green fluorescent protein expression using the transcriptional and translational fusion with the *AGT1* promoter and open reading frame, respectively, revealed that *AGT1* expressed constitutively in all *in vitro* grown cell types and during *in planta* colonization, and localized in peroxisomes. Peroxisomal localization was further confirmed by colocalization with red fluorescent protein fused with the peroxisomal targeting signal 1. Surprisingly, conidia produced by the Δ*agt1* mutant were unable to form appressoria on artificial inductive surfaces, even after prolonged incubation. When supplemented with nicotinamide adenine dinucleotide (NAD^+^)+pyruvate, appressorium formation was restored on an artificial inductive surface. Taken together, our data indicate that AGT1-dependent pyruvate formation by transferring an amino group of alanine to glyoxylate, an intermediate of the glyoxylate cycle is required for lipid mobilization and utilization. This pyruvate can be converted to non-fermentable carbon sources, which may require reoxidation of NADH generated by the β-oxidation of fatty acids to NAD^+^ in peroxisomes. Therefore, it may provide a means to maintain redox homeostasis in appressoria.

## Introduction

*Magnaporthe oryzae* is a heterothallic, haploid ascomycete fungus that causes rice blast, one of the most devastating diseases of rice (*Oryza sativa*) worldwide [1], [2]. This pathogen employs a bi-phasic hemibiotrophic infection strategy to colonize host plants. Infection is initiated through attachment of the conidial tip to the host leaf surface with the help of mucilage. Conidia germinate immediately and germ tubes differentiate into specialized dome-shaped infection structures called appressoria. Following appressorium formation, the internal appressorial turgor pressure essential for mechanical penetration increases dramatically due to the accumulation of glycerol and reaches up to 8 MPa, the highest pressure ever known in any biological system. This facilitates the piercing of the rice cuticle through penetration pegs, which form at the base of appressoria, and become internalized within epidermal cells [3], [4]. Then, the fungus develops bulbous, branched biotrophic invasive hyphae that are encased in an extra-invasive hyphal membrane derived from the host cell plasma membrane, similar to the extra-haustorial membrane developed by obligate biotrophic pathogens [5]. Epidermal cells subsequently become filled with the biotrophic hyphae. After asymptomatic proliferation for up to 3 days, these biotrophic invasive hyphae switch to the necrotrophic phase associated with the production of necrotrophic invasive hyphae, which spread into neighboring cells, causing typical necrotic lesions. Thousands of conidia are produced from these lesions and are released to infect new plants [6].

The infection process of the pathogen, from conidial germination to appressorial penetration, is fuelled by storage reserves carried in the spores. *M. oryzae* conidia are equipped with storage reserves in the form of trehalose, glycogen and lipid bodies (LBs) [7]. Trehalose is metabolized during conidial germination, and rapid glycogen degradation occurs at the same time. LBs are transported to the germ tube apex and eventually to the developing appressorium. Mobilization of LBs is regulated by the Pmk1 mitogen-activated protein kinase (MAPK) pathway [8]. Subsequently, LBs coalesce and are taken up by vacuoles through a process that resembles autophagocytosis [9]. Lipid degradation or lipolysis, which requires cAMP-dependent protein kinase A (PKA), occurs in vacuoles that also coalesce to form a large central vacuole within the maturing appressorium [7].

Appressorium development is tightly coupled with lipid utilization. This can be divided broadly into two different stages: the initiation of appressorium development, which requires cAMP signaling, and appressorium morphogenesis, which requires the presence of the Pmk1 MAPK pathway. The subsequent breakdown of lipids within the appressorium requires cAMP-dependent PKA [7]. A consequence of lipolysis in the appressorium is the generation of both fatty acids and highly soluble osmolyte glycerol. Therefore, a requirement for fatty acid β-oxidation and subsequent activation of the glyoxylate cycle and gluconeogenesis has been proposed [7], [9]. The β-oxidation of fatty acid is a well conserved metabolic process that results in the degradation of fatty acids into acetyl-CoA and reducing equivalents (FADH_2_ and NADH). In yeast, β-oxidation occurs only in peroxisomes [10], [11], [12]. The resultant acetyl-CoA is then transported from peroxisomes to the cytoplasm via carnitine acetyl transferase (Cat) 2 and eventually to mitochondria for oxidation via Cat1 for complete oxidation to CO_2_ and H_2_O through the tricarboxylic acid cycle [13], [14].

We report here that *M. oryzae AGT1*, encoding an alanine: glyoxylate aminotransferase, is expressed constitutively in all fungal developmental stages and during *in planta* colonization, and AGT1 is localized in peroxisomes. Targeted deletion of the *AGT1* resulted in blockage of infection-related appressorial morphogenesis. Furthermore, the Δ*agt1* mutants were unable to form appressoria on an artificial inductive surface, whereas they produced aberrant appressoria on the plant surface. Exogenous addition of pyruvate along with NAD^+^ restored appressorium formation on plastic cover slips. The data obtained in the study suggest that the coordination of β-oxidation and the glyoxylate cycle via AGT1 is required for *M. oryzae* pathogenesis on its hosts rice and barley.

## Results

### Identification of alanine: glyoxylate aminotransferase 1 in *M. oryzae*

Using a complete amino acid sequence, we identified the ortholog of *Saccharomyces cerevisiae* alanine: glyoxylate aminotransferase (EC 2.6.1.44) AGX1 in the *M. oryzae* 70-15 genome (http://www.broadinstitute.org/annotation/genome/magnaporthe_grisea). MGG_02525 open reading frame (ORF; GeneBank accession no. BM863694) was predicted to encode a 386-aa protein, showing 47% identity to AGX1 (Figure S1) and designated as alanine: glyoxylate aminotransferase 1 (AGT1). AGX1 is one of three enzymes required for glycine biosynthesis in *S. cerevisiae*. AGX1 uses pyridoxal 5-phosphate as a co-factor and transfers the amino group of alanine to glyoxylate, thus forming glycine and pyruvate [15].

### *AGT1* replacement mutants are non-pathogenic

**Figure 1:**
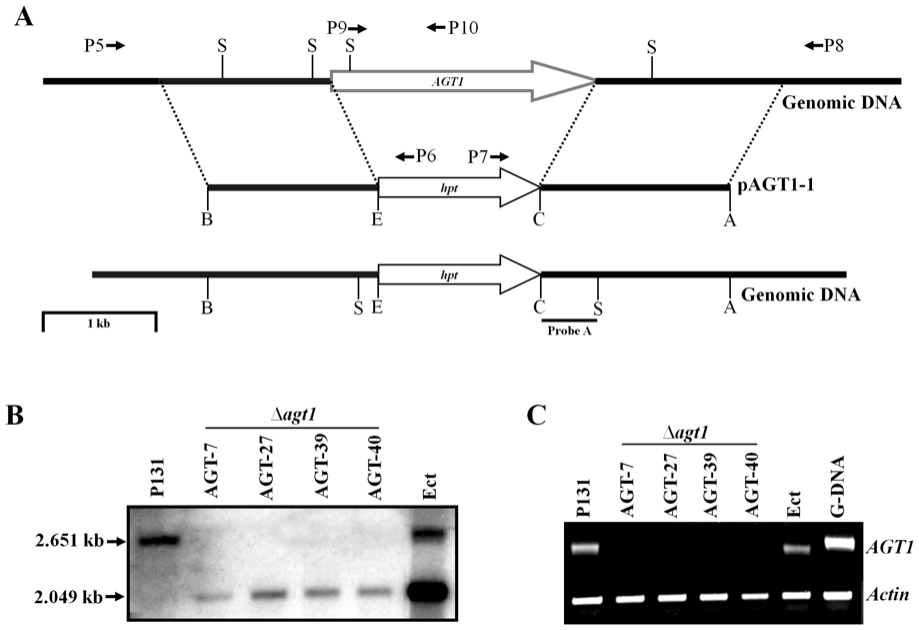
Targeted replacement of the gene *AGT1*. (A) The gene *AGT1* and gene replacement vector pKOV21-AGT1. The gene replacement vector was constructed by replacing the entire *AGT1* locus with the hygromycin phosphotransferase gene (*hpt*). The positions of primers from P5 to P10 used to confirm transformants by PCR are indicated by arrows. A, *Apa*I; B, *Bam*HI; C, *Cla*I; E, *Eco*RI; and S, *Sma*I. (B) Southern blot of SmaI digested genomic DNA of the wild type strain P131, *Δagt1* and ectopic mutants was hybridized by probe A. (C) RT-PCR analysis of *Actin* and *AGT1* (P9/P10) genes. Total RNA was isolated from mycelia of the wild type strain, *Δagt1* and ectopic mutants and converted into cDNA. Thirty-five cycles were used to amplify *AGT1* transcripts.

To characterize the function of *AGT1* in *M. oryzae*, a gene replacement vector pAGT1-1 was constructed by replacing the *AGT1* ORF with the hygromycin phosphotransferase (*hpt*) gene. The *Not*1-digested pAGT1-1 was transformed into the wild-type *M. oryzae* strain P131 (Figure 1A). Forty-five hygromycin B- resistant transformants were isolated and screened by PCR. Four *AGT1* deletion mutants were confirmed by Southern blot analysis (Figure 1B). When probed with a 0.48-kb fragment obtained by digesting a 1.64-kb downstream flanking sequence of *AGT1* with *Cla*I and *Sma*I, the Δ*agt1* mutants AGT-7, AGT-27, AGT-39 and AGT-40 had a 2.0-kb band instead of the wild-type 2.6-kb band. Ectopic integration transformant (Ect) had both mutant and wild-type bands. Null mutation in the knock-out mutants was further confirmed by RT-PCR. No *AGT1* transcript was detected in the Δ*agt1* mutants, whereas a transcript band was present in the P131 and Ect strains, indicating the abrogation of *AGT1* transcripts in knock-out mutants (Figure 1C).

**Figure 2:**
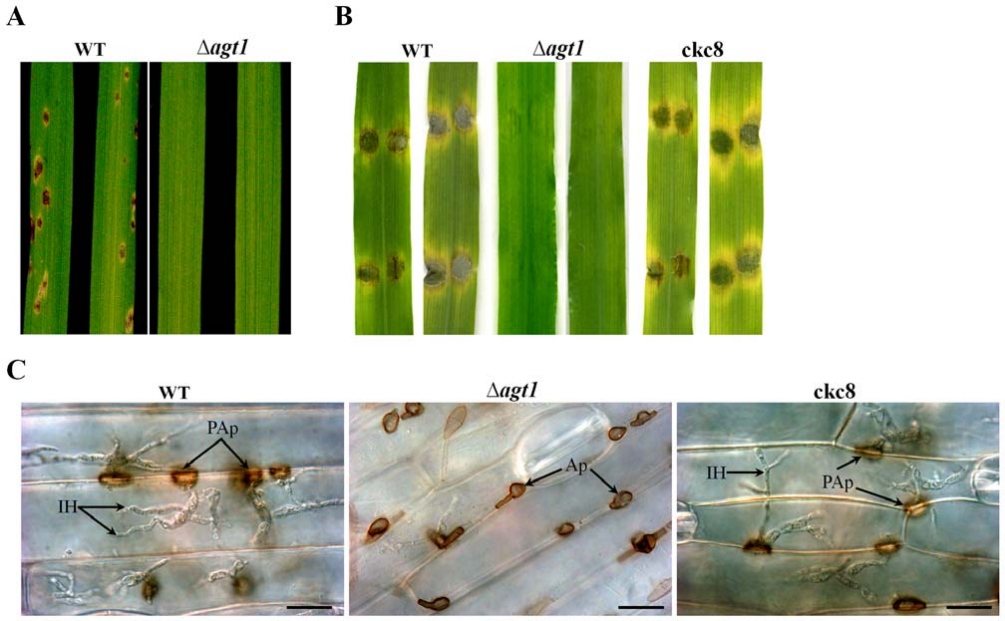
Plant infection assays and microscopic evaluation of infection process. (A) Susceptible rice cultivar Ribenqing was spray-inoculated with conidial suspension (4×10^4^ conidia/mL) of the wild-type P131 and Δ*agt1* strains. Photographs were taken 9 days post inoculation (dpi). (B) Detached leaves of susceptible barley cultivar CDC Silky were droplet-inoculated with P131, Δ*agt1* and complementation (ckc8) strains. Fifteen microliters of conidial suspension (4×10^4^ conidia/mL) were spotted onto the adaxial side of leaves without damaging the surface and photographed 7 dpi. (C) Microscopic observation of droplet inoculated areas of barley leaves 3 dpi. Ap, unpenetrarted appressoria; PAp, penetrated appressoria; and IH, invasive hyphae. Bars = 20 µm.

Targeted deletion of *AGT1* had no observable impact on hyphal elongation or mycelial growth. There was, however, a 19-fold-reduction in conidiogenesis on oat tomato agar (OTA) plates compared to the isogenic strain P131 (4.23×10^7^ and 2.17×10^6^ conidia/ Petri dish [Ø 5.2 mm], respectively in P131 and Δ*agt1*). In order to assess the ability to cause blast disease, we spray-inoculated 4-week-old susceptible rice cultivar Ribenqing with a uniform concentration of the Δ*agt1* mutant and P131 conidia (1×10^4^ conidia/mL), and the plants were allowed to develop blast symptoms for nine days. Rice seedlings inoculated with P131 conidia showed eye-shaped grey centered reddish-brown necrotic lesions (Figure 2A). On the other hand, the Δ*agt1* mutant failed to infect the host, and no blast symptoms were observed on rice leaves. We also conducted infection assays on barley leaf explants of susceptible cultivar CDC Silky. Barley leaves were droplet-inoculated with uniform concentrations of P131 and Δ*agt1* conidia (1×10^4^ conidia/mL). By 7 days post inoculation (dpi), leaf tissues underlying P131 conidial droplets developed water-soaked lesions and collapsed. Under similar conditions, the leaf areas underneath Δ*agt1* conidial droplet remained healthy (Figure 2B). To further confirm whether deletion of the *AGT1* was responsible for the loss of pathogenicity, a gene complementation vector pAGT1-2 was constructed in which *AGT1* was under the control of its native promoter P_AGT1_. The pAGT1-2 vector contained the neomycin resistance gene *nptII*. The *Not*1-digested pAGT1-1 was transformed into the Δ*agt1* mutant, and all 12 isolated neomycin-resistant transformants were similar to the strain P131 in conidiogenesis, conidium germination, appressorium formation and penetration, and invasive *in planta* growth. The complementation strain ckc8 ectopically expressing the wild-type *AGT1* was as virulent as P131 (Figure 2B). These data indicated that the *AGT1* complemented all aberrant phenotypes of the Δ*agt1* mutant. Based on the targeted gene deletion, gene complementation and pathogenicity assays on rice and barley, we conclude that *AGT1* is a key regulator of pathogenesis in *M. oryzae*.

### Loss of *AGT1* leads to production of aberrant appressoria and appressorial penetration failure

Conidia produced by the Δ*agt1* mutants were normal in conidial germination and appressorium formation on plant leaf surfaces. However, the morphology of appressoria differed from those produced by the P131 strain. The vertical and horizontal dimensions of Δ*agt1* appressoria were 6.44±1.05 µm and 15.40±2.22 µm as compared to 9.40±1.23 µm and 12.02±1.74 µm for appressoria formed by P131.

On barley and rice leaves, appressorial penetration was blocked in the Δ*agt1* mutants. By 3 dpi, 84.11±1.20% of the P131 and 83.48±1.20% of the ckc8 appressoria were associated with successful host cell penetration and the development of the invasive hyphae. Under similar conditions, the Δ*agt1* appressoria were unable to initiate penetration of barley and rice leaves, as a result Δ*agt1* mutants failed to cause blast symptoms (Figures 2A, 2B and 2C).

### The Δagt1 mutants are unable to form appressoria on artificial inductive surfaces, whereas exogenous addition of pyruvate+NAD^+^ restores the appressorium formation

**Figure 3:**
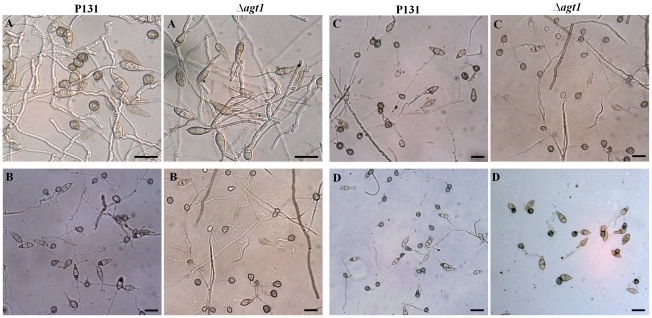
Restoration of appressorium formation of *Δagt1* mutants on artificial inductive surface. Aliquots of 15 µL of conidial suspension (1×10^4^ conidia/mL) of P131 and Δ*agt1* strains supplemented with H_2_O (A), 5 mM NAD^+^ (B), 5 mM NAD^+^+5 mM Pyruvate (C) and 5 mM cAMP (D) were incubated on plastic cover slips and allowed to form appressoria. Bars = 20 µm.

In an *in vitro* assay conducted on plastic slides, conidia produced by the Δ*agt1* mutants formed germ tubes. Occasionally, branching of germ tubes was also observed. Even after prolonged incubation up to 3 days, no hooking at the germ tube tips or appressorium formation was observed. In contrast, conidial germination and appressorial formation (93±2%) was normal in the P131 strain (Figure 3A). However, no significant differences in both developmental processes of the *AGT1* mutant and P131 were found on rice and barley leaves, and on onion epidermis.

To restore the wild-type phenotype of the Δ*agt1* mutants on an artificial inductive surface like plastic cover slips, we conducted a range of *in vitro* pharmaceutical assays. We used a range of concentration gradients of pyruvate and glycine to restore the wild-type phenotype, however, none of them resulted in induction of appressorium formation on artificial surfaces. In contrast, supplementing conidial suspension with 5 mM NAD^+^ restored appressorium formation on plastic cover slips, albeit delayed by 24 h compared to the P131 strain (Figure 3B). When supplemented with pyruvate (5 mM) along with NAD^+^ (5 mM), 92±2% of appressorial formation similar to P131 was observed, demonstrating that *AGT1* indeed encoded alanine: glyoxylate aminotransferase (Figure 3C).

The role of cAMP in stimulating appressorium formation on non-inductive surfaces is well established. Molecular studies have confirmed the role of cAMP signaling in surface recognition and initiation of appressorium formation [16], [17]. To investigate whether cAMP can restore the Δ*agt1* phenotype, conidial suspensions of the P131 and Δ*agt1*strains supplemented with 5 mM and 10 mM cAMP were incubated on plastic cover slips in moist Petri dishes and allowed to form appressoria. Both concentrations of cAMP restored the Δ*agt1* phenotype, indicating that the intracellular level of cAMP in mutants were reduced and therefore, were responsive to exogenous cAMP (Figure 3D).

**Figure 4:**
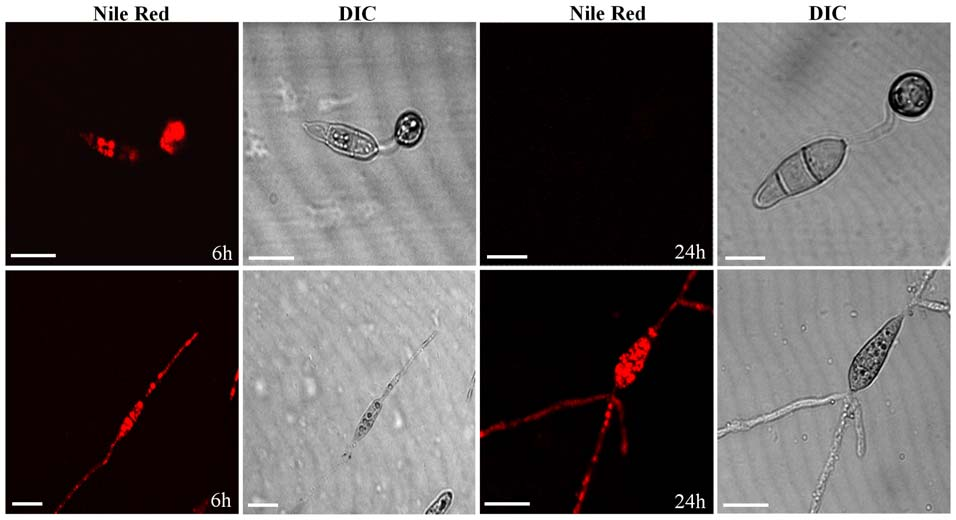
Distribution of lipid droplets during conidial germination and appressorium morphogenesis. Conidia of P131 (upper panel images) and *Δagt1* mutant (lower panel images) were incubated in 15 µL distilled water droplets on plastic cover slips and allowed to form appressoria. Water was removed with a filter paper at 6 and 24 h after incubation, and the conidial layer was stained with Nile red for the presence of triacylglycerol, visualized with confocal microscopy. Bars = 10 µm.

To demonstrate the effect of AGT1 on lipid mobilization and utilization, conidia were allowed to germinate on plastic cover slips and stained with Nile red solution at 6 h and 24 h intervals to visualize triacylglycerol. LBs were predominantly present in appressoria of P131 6 h after incubation (hai). After 24 hai, LBs were depleted at the onset of turgor generation. In contrast, large numbers of LBs remained in conidia of a Δ*agt1* mutant even after 24 hai (Figure 4). The inability to form appressoria by the Δ*agt1* mutant on an artificial inductive surface hampered further investigation. However, LB mobilization during conidial germination and depletion during appressorium maturation was restored in the Δ*agt1* mutant when supplemented with pyryvate+NAD or cAMP.

### *AGT1* is expressed constitutively

**Figure 5:**
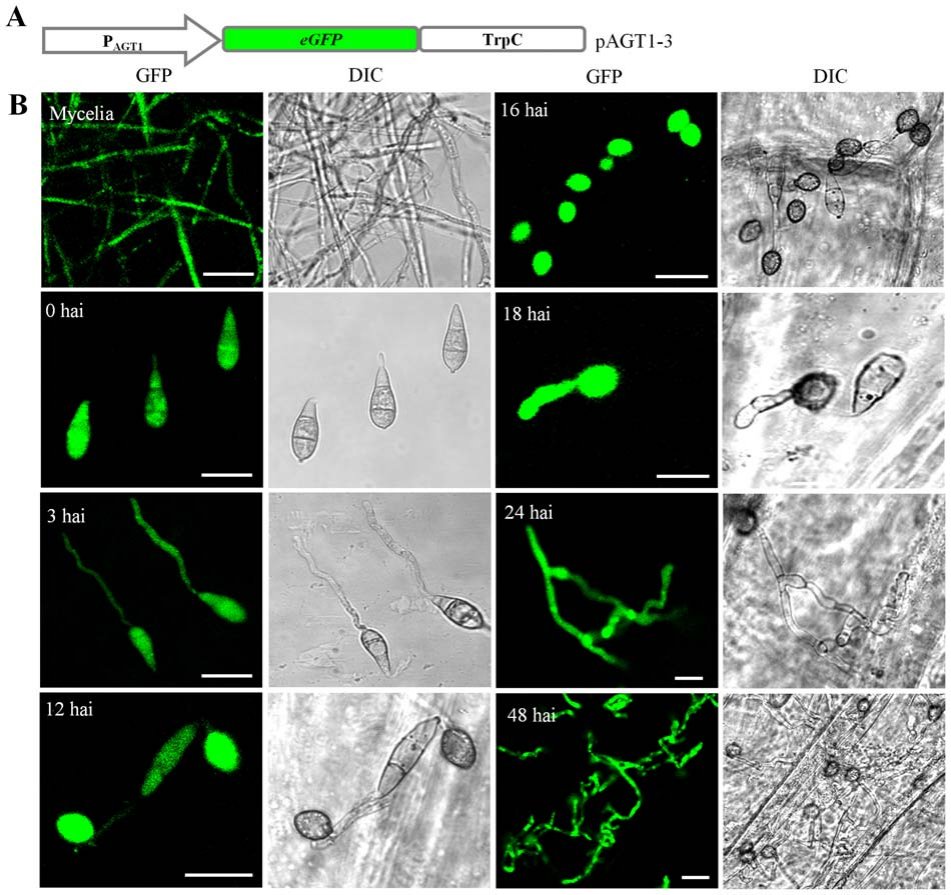
Expression of *AGT1*. Transcriptional activity of *AGT1* was evaluated by incubating or inoculating conidia of P131 transformants expressing the pAGT1-3 construct (eGFP expression was under the control of *AGT1* native promoter) at various stages of fungal development and during *in planta* infection stages. (A) Transcriptional reporter construct. (B) Expression of *AGT1* on an artificial inductive surface (plastic cover slips; 0 and 3 hai) and on yield surface (onion epidermis; 12, 16, 18, 24 and 48 hai). Bars = 20 µm.

To determine the expression pattern and promoter activity of *AGT1*, a 0.52-kb fragment of the *AGT1* promoter (P_AGT1_) was amplified and cloned into the vector pKNTG. The resulting plasmid pAGT1-3 containing the P_AGT1_-eGFP construct (Figure 5A) was used to transform the P131 strain. Ten neomycin-resistant transformants were identified by PCR to contain the P_AGT1_-eGFP construct. In these transformants, no obvious changes in growth or virulence were observed. eGFP signals were detected in the cytoplasm of vegetative hyphae, conidia, germ tubes, appressoria, penetration pegs and invasive hyphae (Figure 5B). These data suggested that the *AGT1* promoter may be constitutively active in *M. oryzae*. By searching the *M. oryzae* EST databases, we found that *AGT1* transcripts were present in cDNA libraries constructed from mycelium, germinated conidia and appressoria [18].

### *AGT1* is localized in peroxisomes

**Figure 6:**
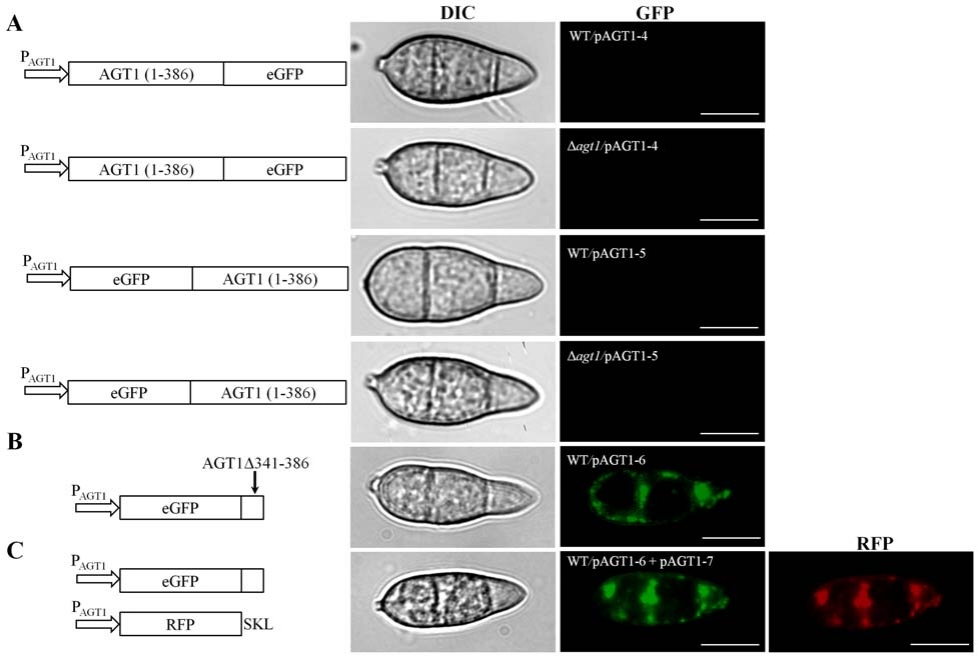
Subcellular localization of the AGT1-eGFP fusion protein. The full length *AGT1* ORF was fused to N- or C-terminus of eGFP (pAGT1-4 and pAGT1-5). In all cases, the translational reporter fusion construct was under the control of *AGT1* native promoter (P_AGT1_). (A) pAGT1-4 and pAGT1-5 were transformed into the wild type P131 and Δ*agt1* strains. Conidia were harvested from 10-day-old oat tomato agar (OTA) plates and examined under a confocal microscope. No eGFP signal was detected in transformants expressing eGFP-AGT1 and AGT1-eGFP. (B and C) Partial AGT1^340-385^ sequence was fused to the C-terminus of eGFP (pAGT1-6) and transformed into the P131 strain. To confirm the peroxisomal localization of AGT1, pAGT1-6 was cotransformed with RFP-PTS1 fusion construct pAGT1-7 into the P131 strain. Conidia harvested from OTA plates of transformants expressing pAGT1-6 and pAGT1-7 showed eGFP fluorescence in a punctuate distribution pattern at the peripheral regions, which was overlapped with RFP fluorescence. Bars = 10 µm.

We generated an *AGT1*-eGFP fusion construct pATG1-4 under the control of its native promoter P_AGT1_. After transforming this construct into the Δ*agt1* mutant, a total of 17 transformants complemented all Δ*agt1* mutant phenotypes. Unfortunately, none of the transformants had detectable eGFP signals in any *in vitro* cell types (vegetative hyphae and conidia), and during *in planta* developmental stages (germination tubes, appressoria and invasive hyphae) (Figure 6A; data shown for conidia). To determine whether the *AGT1* C-terminal fusion might interfere with the detection of eGFP, we generated an eGFP-*AGT1* fusion construct pAGT1-5 and transformed it into the Δ*agt1* mutant. All 20 resulting transformants restored the wild-type phenotype, but eGFP signals were not detectable at various *in vitro* and *in planta* fungal developmental stages (Figure 6A; data shown for conidia). We also transformed pAGT1-4 and pAGT1-5 vectors into the P131 strain. None of isolated transformants showed eGFP fluorescence signals (Figure 6A; data shown for conidia). These results indicated that fusion of *eGFP* to either the N- or C-terminus of *AGT1* had no effect on its function. However, the accumulation of the eGFP fusion proteins may be too low to be detected by confocal microscopy in these transformants. This could be caused by either the expression level of the *AGT1*-eGFP or *AGT1-eGFP* construct, or the stability of fusion proteins. Fusion of the full-length ORF of *AGT1* may have an adverse effect on the proper folding of eGFP.

As the AGT1 protein contained a putative peroxisome targeting signal 1 (PTS1) QKA at the extreme C-terminal end, we also generated the eGFP- AGT1^340–385^ construct (pAGT1-6) and transformed into the P131 strain. The transformants had eGFP fluorescence in a punctuate distribution pattern during all fungal developmental stages (Figure 6B; data shown for conidia). These results indicated that *M. oryzae* AGT1 is located in peroxisome-like bodies (PLB), which are often at the periphery of cells, and that fusion of the C-terminal portion of *AGT1* with eGFP, unlike fusion with the full-length gene, had no effect on fluorescence and lead to PLB location of the fusion protein. Peroxisomal matrix proteins are encoded by nuclear genes, synthesized in the cytoplasm, and then transported into the organelles [19]. Targeting and import of these proteins depend on the peroxisome targeting signals (PTS) in their peptide sequences. PTS can at least be divided into two categories: PTS1 and PTS2 [20]. PTS1, located at the C-terminal of proteins with conserved tripeptide SKL (serine-lysine-leucine) or its derivative (S/C/A-K/R/H-L), is the most abundant motif among all known peroxisomal matrix proteins [21]. PTS2 located at the N-terminal of proteins with a consensus sequence [(R)-(A/L/Q/I)-X5-(H)-(I/L/F)], is found in a few cases [22]. In *M. oryzae*, the SKL is the most frequent PTS1. Therefore, we decided to fuse SKL tripeptide to the C-terminus of the red fluorescent protein (RFP) (pRFP^SKL^) to confirm the cellular distribution of AGT1 in more detail. The pRFP^SKL^ (pAGT1-7) construct was introduced to the same transformant of *M. oryzae* expressing eGFP- AGT1^340–385^. Four single-copy plasmid insert transformants, AGT45-1, AGT45-9, AGT45-15 and AGT45-20, were identified and showed eGFP and RFP fluorescence in a punctuate distribution at exactly the same localization (Figure 6C). Therefore, we conclude that the AGT1 is localized in peroxisomes.

## Discussion

**Figure 7:**
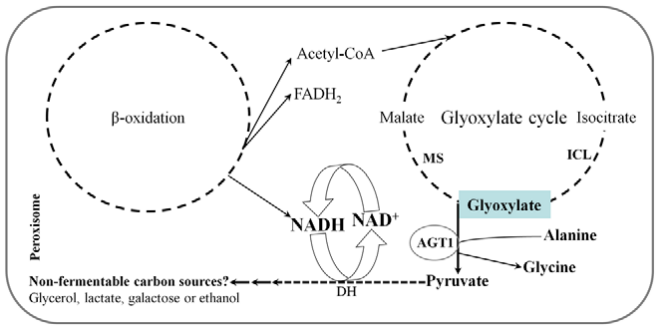
Model of AGT1 mechanism in *M. oryzae*. Peroxisomal oxidation of fatty acids generates the acetyl-CoA and reducing equivalents FADH_2_ and NADH. The acetyl-CoA is channeled through the glyoxylate cycle via isocitrate lyase-mediated production of glyoxylate and the production of pyruvate by AGT1. Reoxidation of NADH to NAD^+^ required for the normal function of β-oxidation and the glyoxylate cycle can be accomplished by the conversion of pyruvate to non-fermentable carbon sources through dehydrogenase (DH) activity. ICL, isocitrate lyase; and MS, Malate synthase.

*M. oryzae* is a model pathogen to study infection-related fungal morphogenesis. This pathogen forms specialized saucer-shaped infection structures known as appressoria, devoted to mechanical penetration of the host plant surface. The mechanical force generated in the appressorium is a consequence of lipolysis of LBs, which are transported from the conidium through the apex of the extending germ tube. As a result, glycerol and fatty acids are produced. The accumulation of molar concentrations of glycerol draws water into the appressorium by osmosis, thereby generating hydrostatic turgor. It allows the fungus to generate an enormous invasive force that is concentrated at the appressorium pore, a central region in contact with the host surface, and through which a penetration peg is forced through the cuticle into the epidermal cell [23]. Fatty acids resulting from lipolysis are transported to peroxisomes where they are metabolized via β-oxidation to form acetyl-CoA and reducing equivalents NADH and FADH_2_, the predominant sources of ATP generation. Acetyl-CoA is then channeled through the glyoxylate cycle via isocitrate lyase (ICL) - mediated production of glyoxylate and the production of malate by malate synthase [24]. In order to maintain lipid mobilization from the conidium and utilization in the appressorium to generate turgor pressure, NADH produced in peroxisomes requires its reoxidation to NAD^+^. This can be accomplished by the production of non-fermentable carbon sources like glycerol, lactate, galactose or ethanol from pyruvate (generated by transferring an amino group of alanine to glyoxylate through AGT1) via dehydrogenase activity, which converts NADH to NAD^+^ and therefore maintains redox homeostasis in peroxisomes (Figure 7).

In this study, we have shown that *M. oryzae AGT1* encodes an alanine: glyoxylate aminotransferase (Figure 3C), and that the rice blast fungus requires a peroxisomal AGT1 for pathogenicity on its host plants rice and barley (Figure 2A and 2B). The *AGT1* deletion mutants failed to induce blast symptoms on host plants, and cytological analysis showed that deletion mutants are impaired in penetrating the host leaf surface (Figure 2C). The inability of Δ*agt1* appressoria to penetrate the plant surface is associated with a defect in LB mobilization to the germ tube apex (Figure 4), which may be the result of perturbations in the NADH/NAD^+^ ratio in peroxisomes due to the continuous generation of NADH via β-oxidation and lack of its reoxidation to NAD^+^ in the absence of AGT1. This blocks the progress of the glyoxylate cycle and as a result, lipid mobilization from the conidium and utilization in the appressorium would be severely impaired, hence Δ*agt1* appressoria fail to generate enough invasive force to penetrate the plant surface. Unlike Δ*agt1* mutants, a delayed appressorium penetration was observed in the null mutants of the gene *ICL1* encoding a glyoxylate cycle enzyme isocitrate lyase [24]. In contrast to the Δ*icl1* mutants, the Δ*agt1* mutants were able to utilize acetate or lipid (olive oil) as a sole carbon source (Figure S2). Intriguingly, unlike on the plant surface, the Δ*agt1* mutants are unable to induce appressorium formation on an artificial inductive surface, suggesting that the hydrophobic plant surface provides additional cues to initiate the appressorium development. However, this defect can be rescued by the exogenous addition of pyruvate in combination with NAD^+^ or cAMP alone. The perturbation in the NADH/NAD^+^ ratio may also affect the intracellular level of cAMP and therefore the Δ*agt1* mutants are responsive to exogenous addition of cAMP and form appressoria on an artificial inductive surface.

In plants, AGT is involved in the photorespiratory glycolate cycle, and is localized in peroxisomes [25], [26]. The situation in mammals is more complex, because species with peroxisomal as well as mitochondrial AGT have been described. In some animals, AGT can be found in both subcellular compartments. The situation has been most extensively studied for human AGT, since mistargeting from the peroxisomes to the mitochondria is involved in the rare disease known as primary hyperoxaluria type 1 (PH1). PH1 is an inherited disease characterized by increased oxalate production from accumulated glyoxylate when AGT is deficient, leading to renal failure [27], [28], [29]. The AGT1 sequence in *M. oryzae* contained a putative PTS1 QKA at the C-terminal end. Nevertheless, while complementing the Δ*agt1* mutant, the eGFP-AGT1 or AGT1-eGFP fusion constructs under the control of the native promoter, failed to produce eGFP signals. As strong green fluorescent signals were detected in transformants expressing the pAGT1-3 construct in which eGFP was under control of the native promoter P_AGT1_ (Figure 5), the promoter activity of 0.52-kb upstream fragment should be sufficient to constitutively express these fusion constructs. Laser scanning confocal microscopy can detect lower expression levels and therefore, it was considered unlikely that the eGFP fusion protein produced any signal. It might be possible that fusion of the full length ORF of *AGT1* had an adverse effect on the folding and fluorescence of the eGFP fusion protein. Supporting this, we could show that when the C-terminal fragment of AGT1 (340–385 residues), containing putative PTS1 (QKA) at the rear end, was fused to the C-terminus of eGFP (pAGT1-6) and expressed in the P131 strain with the native promoter, eGFP signals were observed in a punctuated distribution pattern in mycelia, conidia (Figure 6B), germ tubes, appressoria and invasive hyphae. Transformants generated by co-transformation of pAGT1-6 and pAGT1-7 (RFP-SKL) in the P131 strain showed the overlapping eGFP and RFP signals in punctuated pattern, confirming the peroxisomal localization of AGT1 (Figure 6C). Therefore, our study represents the first report of AGT1 localization in any microbial pathogen.

Taken together, our study indicates that the production of pyruvate in peroxisomes is an important consequence of the glyoxylate cycle, a well conserved metabolic pathway across eukaryotic taxa, which functions in coordination with the β-oxidation through AGT1 and contributes to fungal pathogenicity.

## Materials and Methods

### Strains and growth conditions

The *M. oryzae* field isolate P131 and transformants generated in the current study were maintained at 25°C on OTA plates as described by Peng and Shishiyama [30]. Mycelia collected from 36 h-old cultures in complete medium (0.6% yeast extract, 0.3% acid casein hydrolysate, 0.3% enzymatic casein hydrolysate and 1% sucrose) shaken at 150 rpm were used for protoplast preparation and isolation of fungal DNA. Fungal protoplasts were subjected to PEG/CaCl_2_-mediated transformation as described by Park et al. [31]. The OTA media supplemented with 200 µg/mL hygromycin B (Calbiochem, Germany) and/or 400 µg/mL G418 (Sigma, USA) were used to select resistant transformants. Conidia harvested from 10 day-old OTA cultures were used for conidiation, appressorium formation and penetration assays, and plant infection studies.

### Molecular manipulations with DNA and RNA

Genomic DNA was extracted from vegetative hyphae using the CTAB protocol [32]. Total RNA was isolated using phenol/chloroform extraction and LiCl precipitation [33]. cDNA was prepared using SuperScript II Reverse Transcriptase (Invitrogen, USA). Standard molecular biology procedures were followed for plasmid isolation, DNA gel blot analyses and enzymatic manipulation with DNA [34]. Probes were labeled with the Prime-It II Random Primer Labeling Kit (Stratagene, La Jolla, CA, USA). Plasmid constructs were sequenced with an ABI sequencer (Plant Biotechnology Institute, Saskatoon, Canada). BLAST programs were used to search for homologous DNA and protein sequences. All PCR primers used for constructing vectors and confirming various mutants generated during the study are listed in Table S1.

### Generation of *AGT1* replacement and complementation constructs and mutants

For generating the *AGT1* gene replacement vector pAGT1-1, the 1.42-kb upstream and 1.64-kb downstream flanking sequences of the *AGT1* were amplified with the primer pairs P1/P2 and P3/P4, respectively. The resulting PCR products were cloned into the *Bam*HI-*Eco*RI and *Cla*I-*Apa*I sites of the pKOV21, respectively. The vector pKOV21 was generated by subcloning a 2.1-kb *Sal*I fragment with the bacterial hygromycin phosphotransferase gene (*hpt*) from the plasmid pUCATPH into pKN [35], [36] to provide the neomycin resistance (*nptII*) gene. The *Not*I-digested pAGT1-1 was transformed into P131. Hygromycin-resistant transformants were isolated and subjected to PCR screening using the primer combinations P5/P6, P7/P8 and P9/P10. The primer pairs P5/P6 and P7/P8 were used to amplify 2.3-kb and 1.8-kb fragments, respectively, that are diagnostic for homologous recombination events at the upstream and downstream flanking sequences. The primer set P9/P10 specific to *AGT1* amplified 0.57-kb fragment in the wild type and ectopic integration mutants but not in the Δ*agt1* mutant. Four PCR-confirmed putative Δ*agt1* transformants along with P131 and an ectopic integration mutant ckc8 strains were subjected to Southern blot analysis. RT-PCR analysis was conducted with cDNA synthesized from Δ*agt1*, ckc8 and P131 RNA to confirm the abrogation of *AGT1* expression using the primer set P9/P10.

For generating the complementation construct AGT1-2, the *AGT1* gene along with 0.52-kb upstream sequence was amplified with primer set P11/P12 and cloned between the *Apa*I and *Pst*I restriction sites in the pKNTG vector to generate pAGT1-2. The *Not*I-linearized pAGT1-1 was transformed into the Δ*agt1* mutant. Neomycin-resistant transformants were confirmed by PCR and analyzed for conidiation, appressorium penetration and virulence on barley seedlings.

### Generation of transcriptional and translational reporter constructs

To generate transcriptional reporter construct pAGT1-3, the 0.52-kb promoter region of *AGT1* (P_AGT1_) was amplified from genomic DNA of the strain P131 using P11/P13 and cloned between the *Apa*I and *Cla*I sites of pKNTG. The pKNTG was constructed by cloning the *eGFP*-*Trp*C terminator sequence into pKN [36]. The resulting pAGT1-3 was transformed into the *M. oryzae* wild type strain P131.

The *AGT1*-*eGFP* fusion vector pAGT1-4 was generated by cloning the P_AGT1_ and *AGT1* cDNA into pKNTG using P11/P14 primer set. The *eGFP*-*AGT1* fusion construct, pAGT1-5, was constructed by cloning the *AGT1* ORF between *eGFP* and the *TrpC* terminator of pKNTG using the P15/P16 primer set. The *eGFP-AGT1* ^340–385^ vector pAGT1-6 was constructed by cloning of a 138-bp fragment amplified from the *AGT1* cDNA using the primer pair P16/P17. To confirm peroxisomal localization, we also generated peroxisome targeting vector pAGT1-7 (pRFP^SKL^) by cloning the ORF of the mCherry variant of red fluorescent protein (RFP; GeneBank accession no. AY678264) into pKNTG-HPT (*nptII* was replaced by *hpt*) using the P18/P19 primer set. The PTS1 signal (SKL) sequence was incorporated before the stop codon. All four translation reporter constructs pAGT1-4, pAGT1-5, pAGT1-6 and pAGT1-7 were under control of the native promoter P_AGT1_. pAGT1-4 and pAGT1-5 were transformed into the wild-type strain P131 and Δ*agt1* mutant AGT-40, whereas pAGT1-6 and pAGT1-7 were transformed into the P131.

### Infection assays and cytological assays

Conidia from wild-type and genetically manipulated *M. oryzae* strains were harvested from 10-day-old OTA culture plates and resuspended to 4×10^4^ conidia/mL in sterile water supplemented with 0.025% Tween-20. Four-week-old rice seedlings of cultivar Ribenqing were spray-inoculated and allowed to develop blast symptoms. Photographs were taken 9 dpi. For droplet inoculation, conidial suspension of 4×10^4^ conidia/mL of *M. oryzae* strains were spotted onto adaxial surfaces of detached leaves of 10-day-old seedlings of susceptible barley cultivar CDC Silky without damaging the surface and blast lesions were allowed to develop. Photographs were taken 7 dpi. For microscopic study, barley leaf tissues were collected from droplet-inoculated areas 3 dpi and fixed in a fixation solution (60% methanol, 30% chloroform, 10% acetic acid) until required. Fixed samples were rehydrated with decreasing ethanol gradients (100%, 80%, 70%, and 50% ethanol). Samples were then stained with 0.05% trypan blue (Harleco Parastains, Philadelphia, PA, USA) in distilled water overnight and destained in distilled water. The stained tissues were then mounted in 30% glycerol on glass slides. The developmental stages were examined under a compound light microscope and photographed.

To visualize lipid droplets during conidial germination and appressorium formation under a confocal microscope, conidia collected from P131 and *Δagt1* were stained with Nile red (Sigma, St. Louis, MO, USA) as described previously [7].

### Confocal microscopy

Confocol laser scanning microscopy was performed using a Zeiss Confocor2–LSM 510 (Carl Zeiss, Jena, Germany). eGFP and Nile red, and mCherry RFP were excited with an Argon (488 nm) and HeNe (543 nm) lasers, respectively. Fluorescence signals were captured through the band-pass emission filters 505–530 nm (eGFP) and 600–650 nm (mCherry), and with the long pass emission filter 650 nm (Nile red).

## Supporting Information

Figure S1 **Sequence alignment of MGG_02525 (AGT1;** ***Magnaporthe oryzae*** **alanine-glyoxylate aminotransferase 1) and FL030W (AGX1;** ***Saccharomyces cerevisiae*** **alanine-glyoxylate aminotransferase).** Shaded areas with grey color show conserved amino acid residues.
(TIF)

Figure S2 **The** ***Δagt1*** **mutants are able to utilize acetate or lipid as a sole carbon source.** Strains were grown on minimal growth medium supplemented with sodium acetate or lipid (olive oil). Photographs were taken 7 days after incubation.
(TIF)

Table S1 **PCR primers used in the study.** Primer sequences used for constructing vectors and confirming various mutants generating during the study are listed.
(DOCX)
