# Supplementary figures and images for: Peroxisomal Alanine: Glyoxylate Aminotransferase AGT1 Is Indispensable for Appressorium Function of the Rice Blast Pathogen, Magnaporthe oryzae
Source: PLoS One. 2012 Apr 27;7(4):e36266. doi: 10.1371/journal.pone.0036266 (PMC3338719; doi:10.1371/journal.pone.0036266)

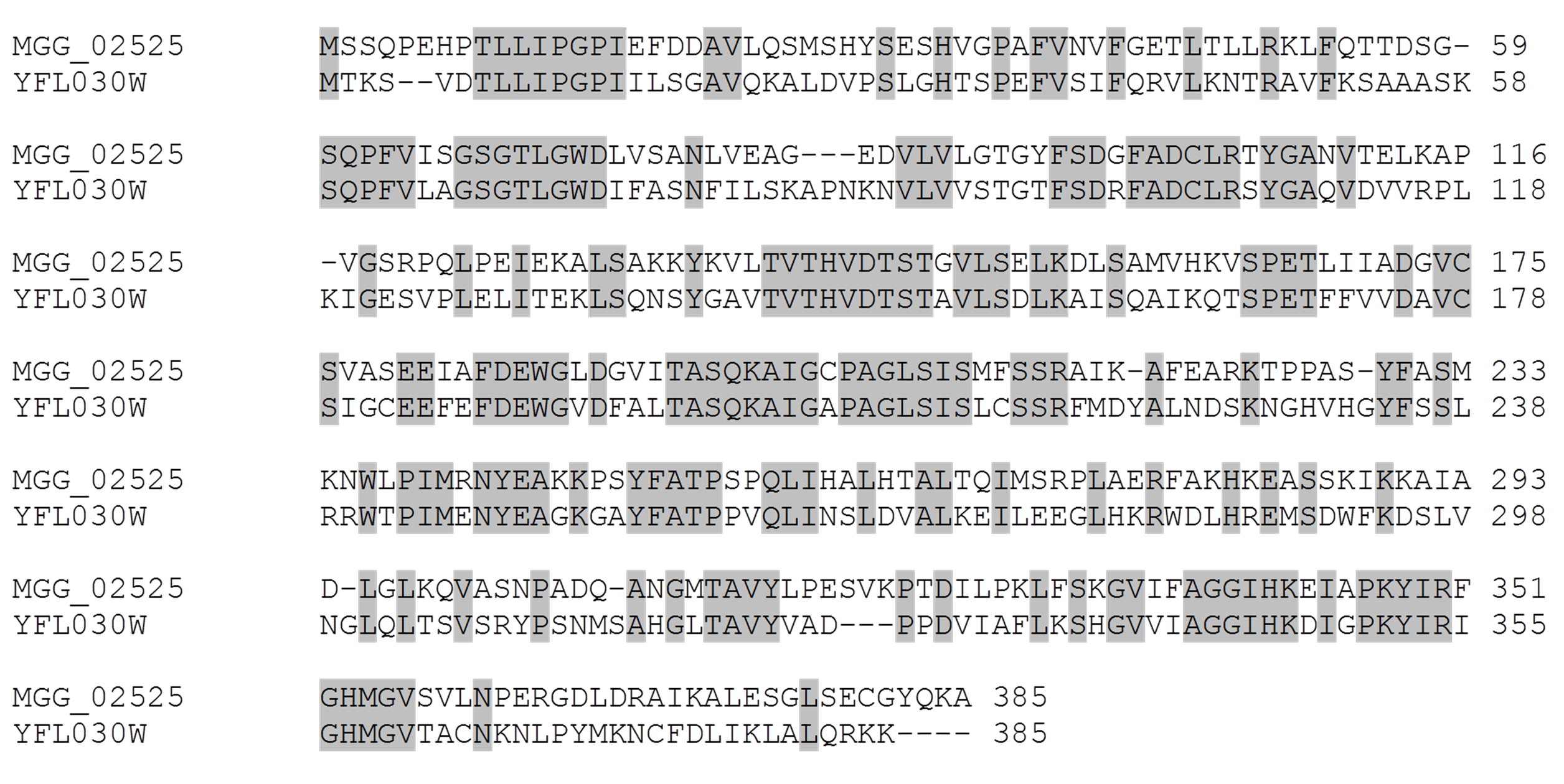

Supplement: Figure S1 — Sequence alignment of MGG_02525 (AGT1; Magnaporthe oryzae alanine-glyoxylate aminotransferase 1) and FL030W (AGX1; Saccharomyces cerevisiae alanine-glyoxylate aminotransferase). Shaded areas with grey color show conserved amino acid residues. (TIF) [file pone.0036266.s001.tif]

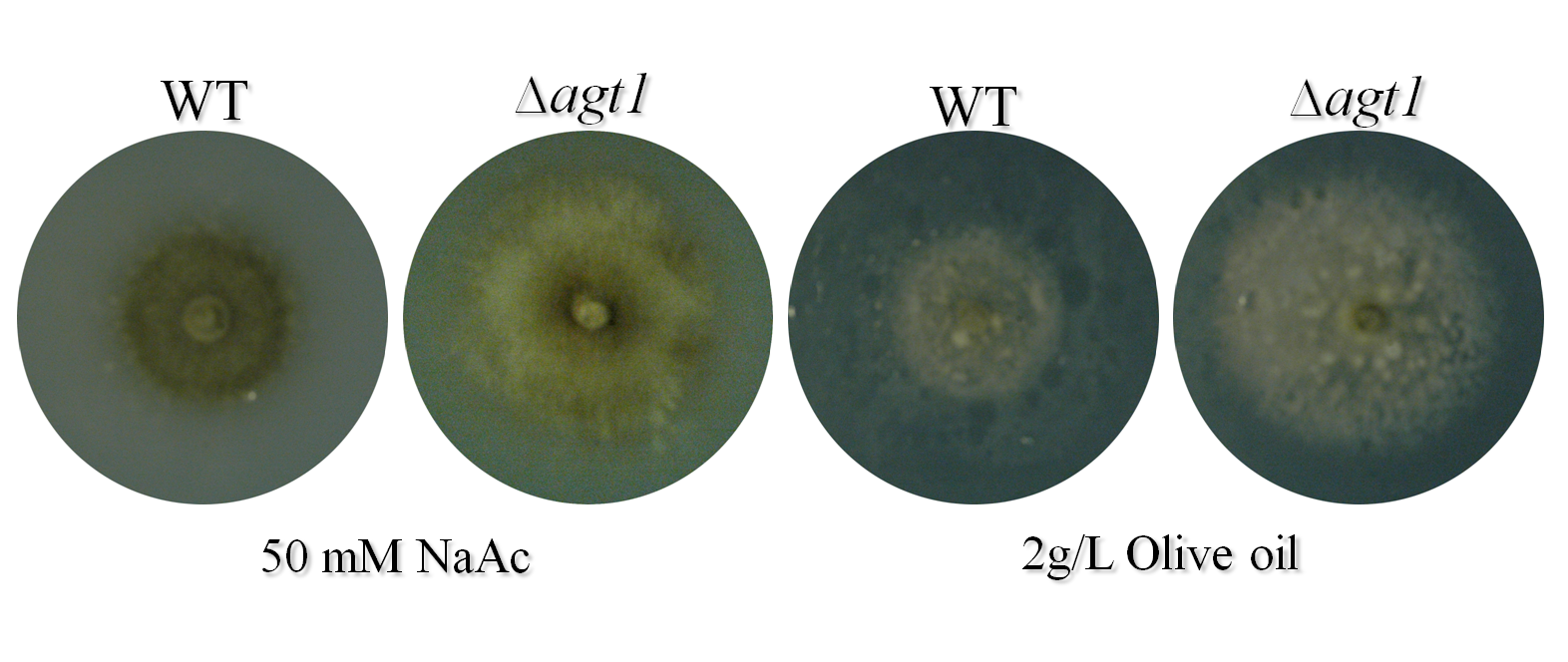

Supplement: Figure S2 — The Δagt1 mutants are able to utilize acetate or lipid as a sole carbon source. Strains were grown on minimal growth medium supplemented with sodium acetate or lipid (olive oil). Photographs were taken 7 days after incubation. (TIF) [file pone.0036266.s002.tif]
