# Supplementary material for: Peroxisomal Alanine: Glyoxylate Aminotransferase AGT1 Is Indispensable for Appressorium Function of the Rice Blast Pathogen, Magnaporthe oryzae
Source: PLoS One. 2012 Apr 27;7(4):e36266. doi: 10.1371/journal.pone.0036266 (PMC3338719; doi:10.1371/journal.pone.0036266)
Supplement: Table S1 — PCR primers used in the study. Primer sequences used for constructing vectors and confirming various mutants generating during the study are listed. (DOCX) [file pone.0036266.s003.docx]

| **Primer** | **Sequence (5’→3’)** | **Vector/**  **Confirmation** |
| --- | --- | --- |
| P1 | GCGGATCCCCAGATGCCGATAGATACC (*Bam*HI) | pAGT1-1 |
| P2 | GAGAATTCACATAATCAAAGCCCACTCC (*Eco*RI) | pAGT1-1 |
| P3 | GCATCGATATCCGATGTGCCTTGTCAACGATG (*Cla*I) | pAGT1-1 |
| P4 | TAGGGCCCAAAGCGTGTTCGCAGAGTGTCG (*Apa*I) | pAGT1-1 |
| P5 | TGGAAGGGCTGGTGTCAAG | Confirmation |
| P6 | GACAGACGTCGCGGTGAGTT | Confirmation |
| P7 | TCTGGACCGATGGCTGTGTAG | Confirmation |
| P8 | CGTCGTAGTTGCTGACTTGTGT | Confirmation |
| P9 | GCTCGGACAATAGCAACAAA | RT-PCR and Confirmation |
| P10 | CCAAGAACCAGCACATCCT | RT-PCR and Confirmation |
| P11 | TAGGGCCCGGGGCCGAAGACGGAGAATT (*Apa*I) | pAGT1-2 (P_AGT1_-AGT1), pAGT1-3 (P_AGT1_-eGFP) and pAGT1-3  (AGT1-eGFP) |
| P12 | GCCTGCAGCTATGCTTTTTGATAACCGC (*Pst*I) | pAGT1-2 (P_AGT1_-AGT1) |
| P13 | GCATCGATTTTGGAATGTTTGTTGCTATTGTCCG (*Cla*I) | pAGT1-3  (P_AGT1_-eGFP) |
| P14 | GCAAGCTTTGCTTTTTGATAACCGCACT (*Hind*III) | pAGT1-4  (AGT1-eGFP) |
| P15 | ATTAGA TCTTATGTCTTCTCAACCTGAGCATCCC (*Bgl*II) | pAGT1-5  (eGFP-AGT1) |
| P16 | AGCGAATTCCTATGCTTTTTGATAACCGCACTC (*Eco*RI) | pAGT1-5  (eGFP-AGT1)  and pAGT1-6 (eGFP-AGT1^340-385^) |
| P17 | ATT*AGATCT*TCACAAGGAGATTGCCCCCAA (*Bgl*II) | pAGT1-6 |
| P18 | GCAAGCTTATGGTGAGCAAGGGCGAGGAGGAT (*Hind*III) | pAGT1-7 (RFP-SKL) |
| P19 | TAGAATTCTTACAGCTTCGACTTGTACAGCTCGTCCATGCCGCC (*Eco*RI; SKL sequence) | pAGT1-7 (RFP-SKL) |
